# Low satisfaction of clients for the health service provision in West Amhara region, Ethiopia

**DOI:** 10.1371/journal.pone.0179909

**Published:** 2017-06-30

**Authors:** Mulatu Melese Derebe, Melashu Balew Shiferaw, Muluken Assefa Ayalew

**Affiliations:** 1 Amhara Public Health Institute, Bahir Dar, Ethiopia; 2 Bahir Dar Health Science College, Bahir Dar, Ethiopia; West Virginia University, UNITED STATES

## Abstract

**Introduction:**

Client satisfaction is a key indicator to measure quality of healthcare and provides information on the level of success forproviders whether client expectations and values are met. Although there are some institutional based studies done in Ethiopia, still client satisfaction in our settings is not well addressed. Thus, this study was aimed to assess client satisfaction level and identify the underlying factors of poor health service provision in West Amhara, Ethiopia.

**Methods:**

A cross-sectional study design was conducted from July to August, 2013. A structured questionnaire was used to collect data from 422 outpatient diagnosis (OPD) service users. The data were entered into EPI Info version 3.5.2 and analyzed usingSPSS version 16.

**Results:**

Among the 422 study participants, 234 (55.5%) males, the mean (±SD) age was 37.3 (±16.4) years. The overall satisfaction level of the study participants was 39.3%. Poor cleanliness of the facility, fewer service access provision, lack of prescribed drugs within the facility and longer waiting time to get the health care service wasreported by 73.2%, 67.8%, 65.6% and 59.2% of the clients respectively. Paying service users (AOR: 2.03, 95% CI: 1.22–3.39, P: 0.007), divorced clients (AOR: 4.26, 95% CI: 1.11–16.26, P: 0.034) and hospital users (AOR: 2.18, 95% CI: 1.29–3.69, P: 0.004) were more dissatisfied.

**Conclusions:**

Client satisfaction was lowin the health provision in West Amhara region. Expansion of health facilities in remote areas, maintaining continuous availability of prescribed drugs, improving cleanliness of health facilities, and fast health service provision are recommended to satisfy clients in the setting.

## Introduction

Client and patient satisfaction become the primary concern to assess the quality of healthcare given and provide information about the level of success of providers to meet client expectations and values. The quality and access of healthcare delivery service can be evaluated according to expected standards [1, 2].

It is obvious that low economic status in developing countries and ill health are inseparable, which mostly fall within the domain of public health systems. Hence, assessing client satisfaction has become a key part of health service management to promote patient oriented health services [3].

Even though Ethiopia strives to meet the clients' satisfaction by implementing the Health Sector Development Program IV (HSDP IV) [4], most clients who used health care facilities complain different health service areas as documented insome literatures that the hospital and health center services had weak healthcare management systems due to poor healthcare service coverage withinsufficient staffing, lack of supplies, inadequate system in infection prevention and high patient flow [5–7]. This indicates that the healthcare system suffers from serious deficiency in quality, efficiency and accessibility. Therefore, improving the healthcare service by using different tools, including efficient E-health technology can guarantee better service to patients.E-health has established connection between medical science and engineering and in this way medical community is able to use engineering facilities such as information technology infrastructure to improve the level of public health. Some of the main reasons of creating electronic health seem to be creating privacy and security for citizens, protection of various cultures and languages, interaction ability between information systems, saving time and money and improving access to the services [8].

Satisfaction manifests itself in factors such as access to services, distribution of health service delivery, and health service utilization.Moreover, data security and privacy are the main challenges as documented in the recent studies [9–11],

Low client satisfaction can reflect the gap between the current experience and the expected servicesthat impact clients to go to more distant public health facilities and even to more costly private health facilities in finding quality healthcare services[12]. It was very much indebted to see what made the clients to be dissatisfied and complain in health care setting to identify major predictors that affect their satisfaction. Although some institutional based studies documented the satisfaction level of clients, still it is not well addressed in our settings.

Hence, this study is intended to make a significant contribution to the existing knowledge of client satisfaction and identify the underlying factors that affect client satisfaction in the healthcare service at government hospitals and health centers in West Amhara region. Lessons learned from this study will be used by the health service strategists to drive up healthcare quality across all service areas.

## Methods

### Study settings

This cross-sectional study was conducted from July 15, 2013 to August 30, 2013 in West Amhara region, which is found in the northern part of Ethiopia.The region had around 10 million people with five zonal districts and two administrative towns. The region shares its border with Benshangul Gumuz regional state in the West and South, Oromiya regional state and Republic of North Sudan in North. The major ethnic groups are Amhara and Awi nationalities. There are 454 government-run health centers. Of which, eight hospitals and 250 health centers are located in urban districts while the remaining belongs to rural districts. These hospitals and health centers are located in strategic areas for easy access for the health service delivery.

### Study subjects and sampling procedure

Patients who visited the selected hospitals and health centers for the health service delivery within the study period were our study population. Every client was included in this study. However, those patients who were not able to respond to the interview due to their serious illness and children who were under 15 years andcame aloneto the health facilities were excluded from the study due to the difficulty of getting consent from them. Four health facilities (Two health centers from South Gondar and Awi zones, and two hospitals from West Gojjam zone and Bahir Dar town administration) were selected using simple random sampling technique.

In order to determine the sample size, 95% confidence level (1-α), 5% absolute precision or expected degree of margin of error (d), and 10% contingency for the non-response were used. The 51.7% client’s satisfaction level was used from a study conducted in Jimma University hospital [3]. Hence, a total of 422 participants from the OPD service users of the hospitals and health centers were selected. Study participants were proportionally allocated to the selected hospitals and health centers according to the number of outpatients.

### Data collection procedures and quality control

Eight trained data collectors who were health care professionals collected the data from the clients in each selected health facility. The data were collected using a structured questionnaire designed with face-to-face interview method(S1 and S2 Files). To ensure data quality,two-days training was provided to supervisors and data collectors about the details of the questionnaire, ethical issues, on how to properly communicate with health facility managers and interview clients.The questionnaire was pre-tested at two healthcare facilities (one hospital and one health center). After the pretest, thequestionnaire was modified based on the identified gaps, and the tool was used to carry out the data collection. The process of data collection was monitored by regular supervision, spot checking and reviewing the completed questionnaires daily.

**Fig 1:**
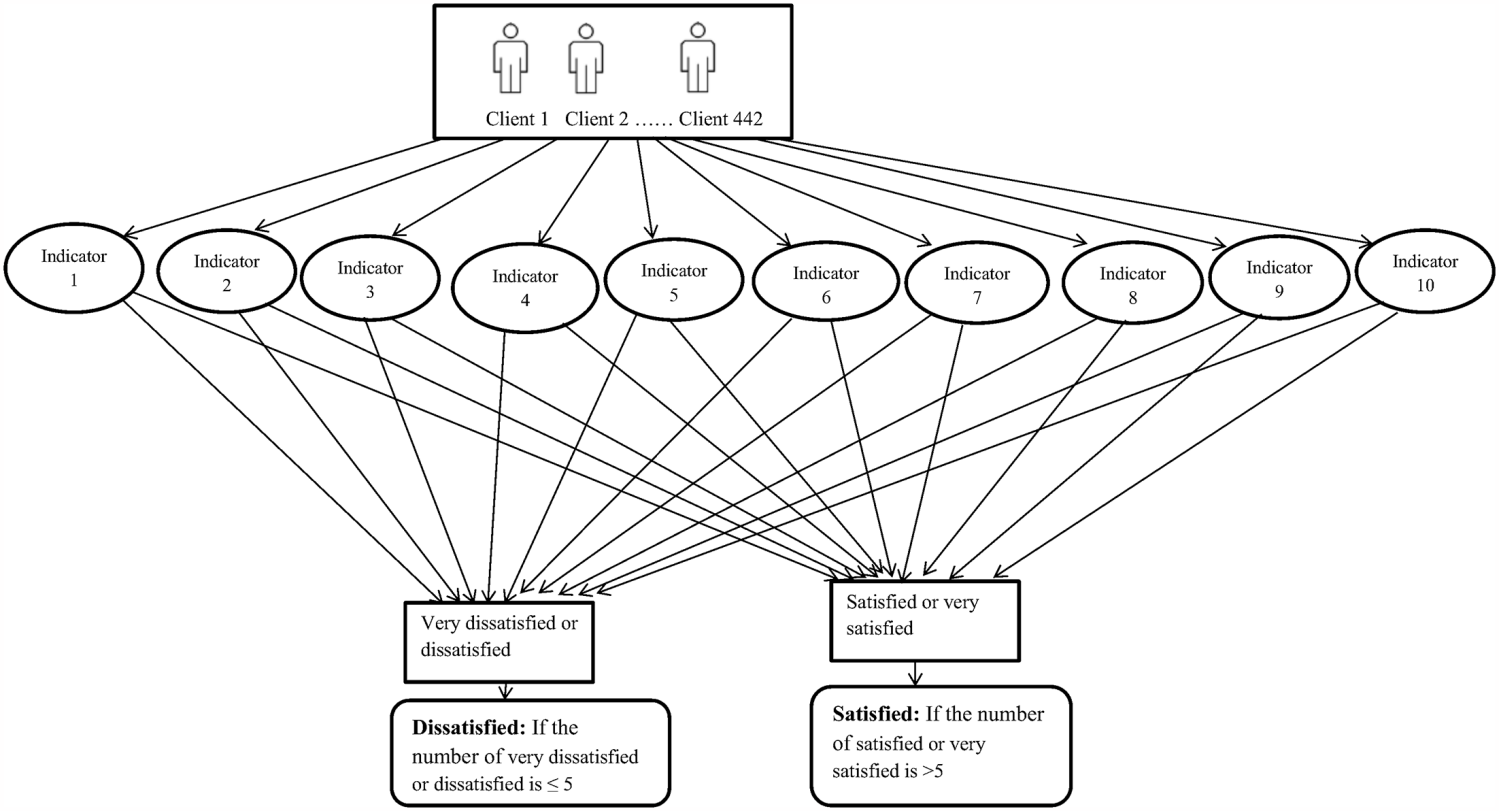
Client satisfaction algorithm and process model. Indicators: 1-Waiting time; 2- Access to service information; 3- Service access; 4- Physical facility; 5- Drug availability; 6- Service cost; 7- Provider-client interaction; 8- Privacy; 9- Examination & consultation; and 10- Cleanness of the facility.

The respondent's data of socio-demographic characteristics and their level of satisfaction with different OPD services were collected properly. Ten indicators of healthcare service measurements (waiting time to receive the service, information provision by the health workers, service accessibility and provision, the physical facility, availability of drugs, treatment cost, health workers and client interactions, privacy of clients, cleanliness of the health facility, and examination and consultation services) were used to evaluate the service quality. The four-point Likert rating scales (very satisfied, satisfied, dissatisfied and very dissatisfied) was used to grade the client’s satisfaction level. Each indicator was rated as very satisfied, satisfied, dissatisfied and very dissatisfied. However, very dissatisfied and dissatisfied responses were considered as dissatisfied while, very satisfied and satisfied were considered as satisfied in order to describe the overall satisfaction level. Accordingly, the ten indicators that influence client satisfaction were includedand rated as either satisfied or dissatisfied in the analysis of overall client satisfaction. One point was given for each indicator if the response had beensatisfied and 0 if unsatisfied. The total score was summed to yield an overall satisfaction score 0 to 10. Thus, clients who responded greater than five scores as satisfied from the ten indicators were rated as satisfied, and those responded ≤ 5 scores were rated as dissatisfied(Fig 1).

### Data analysis

The collected data were entered and cleaned using EPI Info version 3.5.2 and analyzed using SPSS for windows version 16.0 software program. Frequency distribution and percentages were calculated. Variables having P value <0.2 in the bivariate analysis were subsequently tested with multivariate analysis to control confounders. Significant association was considered at P value <0.05 during the final multivariate analysis.

### Ethical consideration

This study was reviewed and approved by the Amhara Regional Health Bureau Research ethics committee followed by written support letter for the health facilities in order to conduct the study. Verbal informed consent was obtained from all the respondents before the start of each interview by explaining the purpose of the study. The exit interviews were conducted where questions and answers did not overhear to ensure privacy and confidentiality of clients. The information obtained from the study was not disclosed to the third body. Only code numbers were used to identify study participants.

## Results

### Socio-demographic characteristics of respondents

Among the 422 study participants, 234 (55.5%) males,the mean (±SD) age was 37.3 (±16.4) years. Larger proportion, 133 (31.5%), of respondents were 45 and above years old.Almost two-thirds of the study participants (67.3%) were married. Two hundred forty three (57.6%) clients were rural residents. Majority of respondents, (81.5%), were paying to get health services. Almost all the study participants (98.6%) visited the health facilities because of their illness (Table 1).

**Table 1:** Socio-demographic characteristics of respondents from health facilities in West Amhara region of Ethiopia, July-August, 2013 (n = 422).

Characteristics		Number	%
Sex	Male	234	55.4
	Female	188	44.6
Age in years	15–24	108	25.6
	25–34	103	24.4
	35–44	78	18.5
	45 +	133	31.5
Marital status	Married	284	67.3
	Single	103	24.4
	Widowed	17	4.0
	Divorced	18	4.3
Religion	Orthodox Christian	401	95
	Protestant	2	0.5
	Muslim	19	4.5
Educational status	Illiterate	251	59.5
	Grade 1–8	75	17.8
	Grade 9–12	58	13.7
	Diploma & above	38	9.0
Residence	Rural	243	57.6
	Urban	179	42.4
Reason for visit	Illness	416	98.6
	Family planning/vaccination	6	1.4
Payment status	Free	78	18.5
	Paying	344	81.5

### Satisfaction of clients in the health service provision

In this study, the overall client satisfaction level was only 39.3%. More than half of the respondents were dissatisfied due to poor cleanliness of the facility (73.2%), fewer service access provision (67.8%) and lack of prescribed drugs within the facility (65.6%). One hundred ninety two (45.5%) and 212(50.2%) of the clients reported the confined OPD rooms and unclean waiting places, respectively. Fifty two (12.3%) of the respondents did not get drinking water, 48 (11.4%) respondents did not have enough latrine in the facility and 73(17.3%) respondents had no proper waiting area with seat.

**Fig 2:**
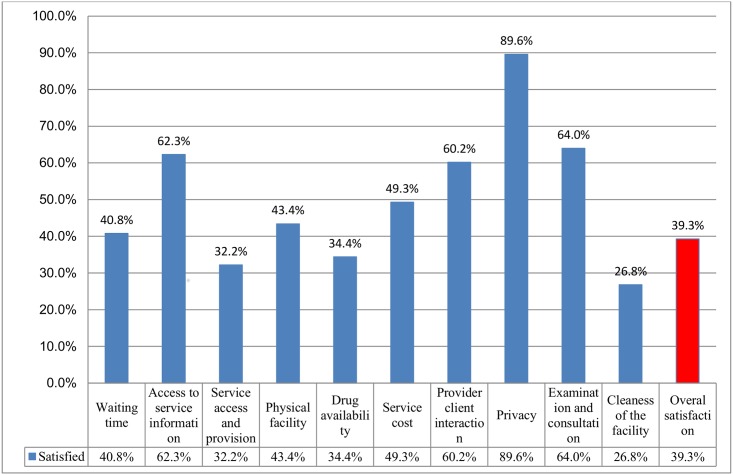
Satisfaction level of clients in health facilities of West Amhara region, July to August, 2013.

Longer total waiting time was reported by 59.2% of the respondents. About 25% of the clients spent greater than 70 minutes to be registered. The clients took greater than two hours to be seen by doctors/clinicians (58 [13.7%]) and to receive the laboratory services (60 [14.2%]). About 37% of the respondentsreached the facility within 2 to 6 hours(Table 2). Respecting the client’s privacy was rated the highest (89.6%) satisfaction level(Fig 2).In the multivariate analysis, clients who were divorced in marital status had 4.26 (AOR: 4.26; 95% CI: 1.11–16.26; P: 0.034) times more dissatisfied than single respondents. Those clients who were free of service charge were 2.03 (AOR: 2.03; 95% CI: 1.22–3.39; P: 0.007) times more satisfied than those who paid for delivered services. Those respondents who used health care service users were 2.18(AOR: 2.18; 95% CI: 1.29–3.69; P: 0.004) times more likely to have a high satisfaction level than hospital users (Table 3).

**Table 2:** Waiting time to get OPD service in health facilities of West Amhara region of Ethiopia, Aug/2013 (n = 422).

Variables	Category	Frequency	Percent
Timespent to be registered	<15 minutes	80	19.0
	15–30 minutes	138	32.7
	31–70 minutes	100	23.7
	>70 minutes	104	24.6
Time to see a clinician	<1 hour	257	60.9
	1–2 hour(s)	107	25.4
	>2 hours	58	13.7
Timespent to receive lab service	<1 hour	262	62.1
	1–2 hour(s)	100	23.7
	>2 hours	60	14.2
Timespent to receive pharmacy service	< 10 minutes	32	7.6
	10–15 minutes	181	42.9
	16–30 minutes	149	35.3
	> 30 minutes	60	14.2
Time spent to see a clinician after lab results	<1 hour	239	56.6
	1–2 hour(s)	136	32.2
	>2 hours	47	11.1
Time to reach the health facility	<1 hour	134	31.8
	1–2 hour(s)	125	29.6
	2–6 hours	163	38.6

**Table 3:** Association of client satisfaction level by socio—Demographic characteristics.

Variables	Category	Satisfied	Dissatisfied	Crude OR	Adjusted OR	P value
Age in years	15–24	40	68	1	-	-
	25–34	49	54	0.65 (0.37–1.12)	-	-
	35–44	33	45	0.80 (0.44–1.46)	-	-
	≥45	44	89	1.19 (0.70–2.03)	-	-
Marital status	Single	43	60	1	1	
	Married	114	170	1.07 (0.68–1.69)	1.10 (0.69–1.76)	0.684
	Widowed	6	11	1.31 (0.45–3.83)	1.83 (0.60–5.59)	0.292
	Divorced	3	15	3.58 (0.98–13.15)	4.26 (1.11–16.26)*	0.034
Edu. status	Illiterate	104	147	0.92 (0.46–1.85)	-	-
	Grade 1–8	23	52	1.47 (0.65–3.33)	-	-
	Grade 9–12	24	34	0.92 (0.40–2.13)	-	-
	Diploma & above	15	23	1	-	-
Payment status	Free	41	37	1	1	
	Paying	125	219	1.94(1.18–3.19)	2.03 (1.22–3.39)*	0.007
Reason to visit	Illness	162	254	3.14(0.57–17.32)	1	
	FP/vaccine	4	2	1	-	-
Frequency of visit	New	98	153	1	-	-
	Existing	68	103	0.97 (0.65–1.44)	-	-
Facility	Hospital	126	224	2.22(1.33–3.71)	2.18 (1.29–3.69)*	0.004
	Health Center	40	32	1	1	-

* Significant association

## Discussion

Client satisfaction is one of the important indicators to measure quality of health care service delivery for the public in Ethiopia [4]. In this study, the overall satisfaction of health provision among clients was 39.3%. This finding is comparable to the studies done in Gondar University hospital [13]and in Tigray zonal hospitals [5]that documented 36.4% and 43.6% of satisfaction level respectively. However, our finding is much lower compared to the 80% of satisfaction targeted at the national level in Ethiopia[4]. It is also lower compared to the 77% satisfaction reported in Jimma University hospital [3], the 62.6% in Central Ethiopia[14], and the 99.6% in Kuwait Primary Health Care[15]. The possible reason which made less satisfaction level of clients in our study might be the difference commitments of regional state health care managers, and the health care providers may also have great effect in designing their strategies to improve health care delivery system, though this may need further study.

In regard of cleanliness of the health facility, only 26.8% of clients were satisfied. Similarly, 27.3% of satisfaction was reported in India[16]. This is lower satisfaction compared to studies conducted in the six regions of Ethiopia (76.5–79.6%) [7]and in Gondar University hospital (65.3%) [13]. This could be due to the inadequate number of cleaners assigned to each service area, poor and confined infrastructure, less awareness of service users regarding cleaning, poor solid waste disposal system and lack of cleaning materials in the facility.

In this study, service accessibility was the second most powerful underlying factor for being clients dissatisfied (distance of health facility to get service, availability of different services, ease to service provision, and availability of competent service providers in all health fields) was rated low as 32.2% satisfied. This satisfaction level is low compared to studies that showed 88.3% of satisfaction in government health facility of India[16]. This may be due to longer distance and lack of convenient transport to reach the facility. Our study showed that 38.6% of respondents reachedto the facility within 2 to 6 hours that needs expansion of more health facilities to the community.

Availability of prescribed drugs within the facility is the most suggested priorities to quality of health care services and treatment outcomes for all service users. However, our study showed that two-third (65.6%) of clients lacked prescribed drugs. In line with this study, Fekadu A. et al reported 70% of unavailability of prescribed drugs in Jimma Hospital[3]. The reason could be associated with increasing number of OPD service users with less correspondence with budget allocation and in adequate health facility reform to avail the necessary drugs based on the clients need.

Our study indicated that 59.2% of clients were dissatisfied by the waiting time to get the health service. This finding is higher when compared with the study conducted in Jimma Hospital by Olijera and Gebre-selassie[17]previously, which was 20.4%, and later it was 37.2% studied by Fekadu A. et al[3]. Likewise, the dissatisfaction level with overall waiting time to receive the service in Nigeria public hospital was rated as 48% [18]and study conducted in India that showed as 35.4% by Kumari et al [16]. This higher level of dissatisfaction rate with over all waiting time could be attributed to the increased number of clients in the health care service, less proportional number of health care providers with clients, and educational background of the study participants to understand that some health care service requires time to provide quality of care.

In our study,the overall client satisfaction in the health centers was significantly higher (55%) than that of hospital services (36%) when compared within their service areas given. Similarly, the clients who used services with payment were more dissatisfied than those service users with free of charge. The reason could be due to high medical service cost and the expectation of the clients to have high quality of service for the cost they incurred.

## Conclusions

The satisfaction of client for the health provision was low in West Amhara region due to poor cleanliness of the facility, fewer service access provision, lack of prescribed drugs within the facility, and longer total waiting time. Hospital service users, divorced clients, and paying patientswere more dissatisfied. Therefore, expansion of health facilities in remote areas, closer follow-up to maintaincontinuous availability of drugs and improving cleanliness the health facility, and fast health service provision are recommended.

## Supporting information

S1 File Questionnaire (English version). (DOCX)

S2 File Questionnaire (Amharic version). (DOCX)
