# Supplementary material for: Low satisfaction of clients for the health service provision in West Amhara region, Ethiopia
Source: PLoS One. 2017 Jun 30;12(6):e0179909. doi: 10.1371/journal.pone.0179909 (PMC5493333; doi:10.1371/journal.pone.0179909)
Supplement: S1 File — (DOCX) [file pone.0179909.s001.docx]

*A Questionnaire to assess outpatient Clients’ and patients’ Satisfaction With the healthcare Services in Hospitals and Health centers*

please kindly fill this interview based questionnaire objectively and clearly that aimed to assess your satisfaction with the provided healthcare service and to identify factors that affect the service in order to improve and enhance the services.

|  | | Date of the questionnaire completed ___/___/___ |
| --- | --- | --- |
| **Hospital/health center:** |  | |
| **Address of clients/patients:** |  | |
| **Completed by:** |  | |

**1. Socio-demographic data**

**Sex** M F **Age** (in years)________

**Residence**- Rural Urban

**Religion -** Orthodox Protestant catholic Muslim others

**Ethnicity -** Amhara Awi Oromo others

**Marital Status**- single married widowed divorced

**Educational level**- Illiterate Grade 1-8 Grade 9-12 Diploma Degree & above

**Occupational status**-Farmer Merchant Government employ Student House wife Others

**Payment status**- Free Paying

**Reason for visit**- Illness Family planning and Vaccination users Others

**Frequency of visit** (within 12 months**)**- New visit Repeat visit

**Health facility**- Hospital Health center

**2. Level of clients' and patients' satisfaction with the different health care services components**

**1. Waiting time to get the service**

- 1. The length of time to open the facility to the public (hrs)_______
  2. Time spent to register (hrs)_______
  3. Time spent to see a Dr (hrs)_______
  4. Time to receive lab service (hrs)_______
  5. Time to receive Pharmacy service (hrs) _______
  6. Waiting time to see the Dr after receiving the x-ray and or lab results? _______
  7. Which service you take long waiting time? _______
  8. How you were satisfied with the overall waiting time to receive whole services?

Very Unsatisfied Unsatisfied Satisfied Very Satisfied

**2. Provision of information by the health professionals**

1. Is there complete Signboard to guide and give you a clear information for each service area? **Yes No**
2. Does health professionals gives you an adequate information about the service process they give? (The Nurses, physicians, pharmacists, Lab personnel) **Yes No**
3. If not, which service providers didn't give you the necessary information?

Nurses , physicians , pharmacists , Lab personnel x-ray Tech

1. How you were totally satisfied with the accessibility to information on the services you received? **Very Unsatisfied Unsatisfied Satisfied Very Satisfied**

**3. Service accessibility**

1. How long time you take to reach the facility?__ hr. Do You fill this is too far for you ?**Yes No .** How much it will be fair to access the facility in your perception? in hr.-------
2. have you got expected full services such as laboratory, Nursing, physician, pharmacy, x-ray? **Yes No**  if No, which service you have received did you dislike?----------------------
3. Did you received a complete Physicians service? **Yes No**  NA
4. Did you received a complete Nursing care? **Yes No**  NA
5. Did you received a complete pharmacy service? **Yes No**  NA
6. Did you received a complete Laboratory service? **Yes No**  NA
7. Did you received a complete X-Ray service? **Yes No**  NA
8. Is all service providers are available and proportional to clients and patients /to you/? **physicians -**Yes No **Nurses-** Yes No **Pharmacists-** Yes No **Lab personnel-** Yes No X-Ray
9. How you were totally satisfied with the service access from the service you have received? **Very Unsatisfied Unsatisfied Satisfied Very Satisfied**

**4. Physical facility**

1. did have proper waiting area with adequate seat? **Yes No**
2. have you get latrine in the facility?  **Yes No**
3. does drinking water available? **Yes No**
4. Have you ease to get bed, medical supplies and equipments for your treatment? **Yes No**
5. Does the building is comfortable to receive the health services? **Yes No**  **if No, Old unclean , Confined**
6. How you were totally satisfied with the physical facility? **Very Unsatisfied Unsatisfied Satisfied Very Satisfied**

**5. Drugs availability**

1. Have you get all prescribed drugs in the facility? **Yes No**
2. How you were totally satisfied with drugs availability in the facility ?

**Very Unsatisfied Unsatisfied Satisfied Very Satisfied**

**6. Treatment cost**

1. How would you rate the cost of services of laboratory, drug and total medication cost

**High Normal Low Don't Know**

1. How you were satisfied with cost of the services ?

**Very Unsatisfied Unsatisfied Satisfied Very Satisfied**

**7. Provider-patient interactions**

1. did the provider made you feel at ease when they receive you? **Yes No**
2. did the provider give attention to listening your complaints? **Yes No**
3. How well were you attended by the health providers at health center/hospital?
   Courteous Impatient Not attentive
4. did the provider explained and discussed well to you about your disease and treatment? **Yes No**
5. How you were totally satisfied with **Provider-patient interactions** during service received? Very Unsatisfied Unsatisfied Satisfied Very Satisfied

**8. Privacy**

1. did the health provider communicate in your language? **Yes No**
2. Did the health provider respect your privacy during consultation? **Yes No**
3. Did your investigation results are disclosed for others without your permission? **Yes No**

**9. Cleanness**

1. Have you get clean waiting area? **Yes No**
2. Have you get clean latrine? **Yes No**
3. Have you get clean outside environment of the health center/Hospital? **Yes No**
4. Have you get clean OPD rooms? **Yes No**

**10. Examination and consultation**

1. Have you get a chance to consult the main doctor/clinician? Yes No
2. Is there a separate place for examination? Yes No
3. Did examination rooms were comfortable for the client/patient? Yes No
4. Did you treated equally with other patients/clients? Yes No
