# Supplementary material for: Low satisfaction of clients for the health service provision in West Amhara region, Ethiopia
Source: PLoS One. 2017 Jun 30;12(6):e0179909. doi: 10.1371/journal.pone.0179909 (PMC5493333; doi:10.1371/journal.pone.0179909)
Supplement: S2 File — (DOCX) [file pone.0179909.s002.docx]

**በምዕ/አማራ በሚገኙ ሆስፒታልና ጤና ጣቢያወች የተገልጋዮችን እርካታ ለመለካት የተዘጋጀ መጠይቅ**

ይህ የተዘጋጀው መጠይቅ በዚህ ጤና ድርጅት እርስዎ ያገኙትን የአገልግሎት እርካታ ለመለካትና አገልግሎቱን ለማሻሻል የሚጠቅሙ ጉዳዮችን ለማወቅና አገልግሎቱን ለወደፊት ለማሻሻል ስለሆነ እባክዎትን ለምጠይቅዎ ጥያቄ በግልጽ እንዲመልሱልኝና እንዲተባበሩን በትህትና እንጠይቃለን?

|  | | መጠይቁ የተሞላበት ቀን _____/___/___ |
| --- | --- | --- |
| የሆስፒታሉ/ጤና ጣቢያ ስም: |  | |
| የተገልጋዩ አድራሻ /ወረዳ/ቀበሌ |  | |
| መጠይቁን የሞላው ባለሙያ ስም: |  | |

**1. የማህበራዊ ገጽታ መረጃ/Socio-demographic data/**

**ጾታ** ወ ሴ **እድሜ** (በዓመት)________

**መኖሪያ ቦታ** - ገጠር ከተማ

**ሃይማኖት -** ኦርቶዶክስ ፕሮቴስታንት ካቶሊክ ሙስሊም ሌላ

**ብሄር -** አማራ አዊ ኦሮሞ ሌላ

**የጋብቻ ሁኔታ**- ያላገባ ያገባ የሞተበት/ባት የተፋታ/ች

**የት/ት ደረጃ**- ያልተማረ 1-8 9-12 ዲፕሎማ ዲግሪና በላይ

**የስራ ዘርፍ**- አርሶ አደር ነጋዴ የመንግስ ሰራተኛ ተማሪ የቤት እመቤት ሌላ

**አገልግሎት የሚያገኘው**- ነጻ ታካሚ ከፍሎ የሚታከም

**የመጡበት ምክንያት**- በህመም ምክንያት ለቤተሰብ ምጣኔ/ለክትባት ለሌላ

**የመጡበት ጊዜ** (በ12 ወራት**)**- አዲስ በድጋሜ የመጣ

**ጤና ተቋሙ**- ሆስፒታል ጤና ጣቢያ

**2. በተለያዩ የጤና አገልግሎቶች በተመላላሽ የደንበኞች የሚካሄድ የአገልግሎት እርካታ ዳሰሳ**

**1. አገልግሎቱን ለማግኘት የሚወስደው ጊዜ /Waiting time to receive the service/**

- 1. ተቋሙ ለህዝቡ አገልግሎት የሚከፈትበት ጊዜ ቆይታ (በስዓት)_______
  2. ካርድ ለማውጣት የሚወስደው የቆይታ ጊዜ (በስዓት)_______
  3. ዶክተሩን ለማግኘት የሚወስደው የቆይታ ጊዜ (በስዓት)_______
  4. የላቦራቶሪ አገልግሎት ለማግኘት የሚወስደው የቆይታ ጊዜ (በስዓት)_______
  5. የመድሃኒት አገልግሎት ለማግኘት የሚወስደው የቆይታ ጊዜ (በስዓት)_______
  6. ከላቦራቶሪና ራጅ ምርመራ በኋላ ዶክተሩን ለማግኘት የሚወስደው የቆይታ ጊዜ (በስዓት)_____
  7. ካገኙት አገልግሎት ውስጥ ረዥም ጊዜ የወሰደብዎት የትኛው ነው? _______
  8. አጠቃላይ አገልግሎቱን ለማግኘት በወሰደብዎት የቆይታ ጊዜ ምን ያህል ረክተዋል?

በጣም አልረካሁም አልረካሁም ረክቻለሁ በጣም ረክቻለሁ

**2. የባለሙያወች መረጃ አሰጣጥ /Provision of information by the health professionals/**

1. እያንዳንዱን አገልግሎት የት ማግኘት እንዳለብዎት የሚጠቁም የመረጃ ሰሌዳ አለ ወይ? **አዎ የለም**
2. ለሰጠዎት አገልግሎት ከባለሙያወች ተገቢውን መረጃ አግንተዋል ወይ? (ከነርሶች፣ ከሃኪሞች፣ ፋርማሲ ባለሙያው ፣ ላቦራቶሪ ባለሙያወች ፣ ራጅ ባለሙያ ) **አዎ የለም**
3. **መልስዎ የለም ከሆነ፤** የትኛው አገልግሎት ሰጭ ነው ተገቢውን መረጃ ያልሰጥዎት?

ነርስ ከሃኪም ፋርማሲ ባለሙያው ላቦራቶሪ ባለሙያው ራጅ ባለሙያ ሌላ

1. በአገልግሎት ወቅት የተሟላና ግልጽ መረጃ ከማግኘት አንጻር ምን ያህል ረክተዋል?

በጣም አልረካሁም አልረካሁም ረክቻለሁ በጣም ረክቻለሁ

**3. የአገልግሎት አሰጣጥና ተደራሽነት /Service accessibility/**

1. ጤና ድርጅቱ ለመድረስ የወሰደብወት ጊዜ ምን ያህል ነው ?____ ስዓት
2. የጤና ድርጅቱ ያለበት ቦታ ለርስዎ ሩቅ ነው ብለው ያምናሉ ? **አዎ የለም**
3. በርስዎ ሀሳብ ጤና ድርጅቱ ምን ያህል ስዓት ቢወስድብዎት ጥሩ ነው ብለው የሚያምኑ? ------ስዓት
4. የሚጠብቁትን ያህል የነርስ፣ሃኪም፣ፋርማሲ፣ላቦራቶሪ፣ራጅ አገልግሎት አግንተዋል ወይ? **አዎ የለም**
5. ከሃኪሙ/ዶክተሩ የፈለጉትን ያህል አገልግሎት አግኘረተዋል ወይ? **አዎ የለም**
6. ከነርሷ/ሱ እንክብካቤ የፈለጉትን ያህል አገልግሎት አግኝተዋል ወይ? **አዎ የለም**
7. ከመድሃኒት ቤቱ የፈለጉትን ያህል አገልግሎት አግኝተዋል ወይ? **አዎ የለም**
8. ከላቦራቶሪ ክፍሉ የፈለጉትን ያህል አገልግሎት አግኝተዋል ወይ? **አዎ የለም**
9. ከራጅ ክፍሉ የፈለጉትን ያህል አገልግሎት አግኝተዋል ወይ? **አዎ የለም**
10. አገልግሎት ሰጭ ባለሙያዎች ለደንበኞች በቂና ተመጣጣኝ ቁጥር አላቸው ብለው ያምናሉ ? **አዎ የለም**  የሃኪሞች/ዶክተሮች ቁጥር በቂ ነው **-አዎ የለም**  የነርሶች **አዎ የለም**  የፋርማሲ ባለሙያወች **አዎ የለም**  የላቦራቶሪ ባለሙያወች **አዎ የለም**  የራጅ ባለሙያወች **አዎ የለም**
11. ባገኙት አጠቃላይ የአገልግሎት አሰጣጥና ተደራሽነት ምን ያህል ረክተዋል?

በጣም አልረካሁም አልረካሁም ረክቻለሁ በጣም ረክቻለሁ

**4. ተቋማዊ ኦፕሬሽናል አገልግሎት /Physical facility/**

1. በጤና ተቋሙ ተስማሚና በቂ መቀመጫ ያለው የመቆያ ቦታ አለ ወይ? **አዎ የለም**
2. በጤና ተቋሙ መጸዳጃ ቤት አግኝተዋል ወይ ? **አዎ የለም**
3. በጤና ተቋሙ ለመጠጥ የሚውል ውሃ አለ ወይ ? **አዎ የለም**
4. ለህክምና አገልግሎትዎ የሚውሉ አልጋ እና ሌሎች የህክምና መሳሪያወችን በቀላሉ በተቋሙ ማግኘት ችለዋል ወይ? **አዎ የለም**
5. ለአግልግሎቱ ተስማሚ ከመሆን አንጻር የጤና ተቋሙ አጠቃላይ ሁኔታ ምን ይመስላል? **አዲስ**  **አሮጌ ንጽህናው ያልተጠበቀ የተጨናነቀ**
6. ባገኙት የህንጻው አገልግሎት በአጠቃላይ ምን ያህል ረክተዋ?

በጣም አልረካሁም አልረካሁም ረክቻለሁ በጣም ረክቻለሁ

**5. የመድሃኒት አገልግሎት /Drugs availability/**

1. በሃኪም የታዘዘልዎትን መድሃኒት በሙሉ በጤና ተቋሙ መድሃኒት ቤት አግኝተዋ ወይ ? **አዎ የለም**
2. መድሃኒት በተቋሙ መድሃኒት ቤት በማግኘትዎ ምን ያህል?

በጣም አልረካሁም አልረካሁም ረክቻለሁ በጣም ረክቻለሁ

**6. የህክመና ወጭ /Treatment cost/**

1. ለአጠቃላይ የህክምና ያወጡትን ወጭ (ላብ፤ መድሃኒትና ሌሎች የህክምና ወጭወችን) በዋጋ ደረጃ እንዴት ያዩታል? **ከፍተኛ**  **ተመጣጣኝ ዝቅተኛ አላውቅም**
2. ለአገልግሎቱ ባወጡት ወጭ ምን ያህል ረክተዋል ?

በጣም አልረካሁም አልረካሁም ረክቻለሁ በጣም ረክቻለሁ

**7. አገልግሎት ሰጭውና ደንበኛው ያለው የግንኙነት ሁኔታ /Provider-patient interactions/**

1. ባለሙያው አገልግሎት ለመስጠት ሲቀበልዎ በደስታ መንፈስ ነው ወይ? **አዎ የለም**
2. አገልግሎት ሰጭው የርስዎን ህመም ለመስማት ትኩረት በመስጠት ነበር ወይ? **አዎ የለም**
3. በባሉያዎች በምን ሁኔታ ተስተናገዱ? **በትህትናና አክብሮት በተሞላበት**  **በማገላመጥና በመናደድ**  **በግዴለሽነት**
4. አገልግሎት ሰጭው ስለ በሽታዎና ህክምናው በሚገባ አስረድትዎታል ወይ? **አዎ የለም**
5. በአገልግሎቱ ወቅት ከባለሙያው ጋር በነበረው ግንኙነት በአጠቃላይ ምን ያህል ረክተዋል?

በጣም አልረካሁም አልረካሁም ረክቻለሁ በጣም ረክቻለሁ

**8. የግል ስብዕና /Privacy/**

1. አገልግሎት የሰጠዎ ባለሙያ በርስዎ ቋንቋ ነው ወይ ያናገርዎት? **አዎ የለም**
2. ባለሙያው በሚያማክርወት ጊዜ የግል ሁኔታዎን በጠበቀ መልኩነበር ወይ? **አዎ የለም**
3. ያለ ርስዎ ፈቃድ የርስዎን ህክምና ውጤት ለሌላ አስተላልፏል ወይ? **አዎ የለም**
4. በህክምና ወቅት የግል ስብእናዎን ከመጠበቅ አንጻር ምን ያህል ረክተዋል?

በጣም አልረካሁም አልረካሁም ረክቻለሁ በጣም ረክቻለሁ

**9. ንጽህና /Cleanness/**

1. ንጹህ የመቆያ ቦታ አግኝተዋል ወይ? **አዎ የለም**
2. ንጹህ መጸዳጃ ቤት አግኝተዋል ወይ? **አዎ የለም**
3. ንጹህ የጤና ተቋም አካባቢ አግኝተዋል ወይ? **አዎ የለም**
4. ንጹህ የህክምና ክፍሎች አግኝተዋል ወይ? **አዎ የለም**
5. በህክምና ተቋሙ ባገኙት ንጹህ የአገልግሎት አካባቢ ምን ያህል ረክተዋል?

በጣም አልረካሁም አልረካሁም ረክቻለሁ በጣም ረክቻለሁ

**10. የህክምና ምርመራና ማማከር /Examination and consultation/**

1. ዋናውን ሀኪም የማግኘትና የማማከር እድል አግኝተዋል ወይ?  **አዎ የለም**
2. ለምርመራ/አገልግሎት የተለየ ክፍል አለ ወይ? **አዎ የለም**
3. የምርመራ/አገልግሎት ክፍሎች ለደንበኞች ምቹ ናቸው ወይ? **አዎ የለም**
4. ከሌሎች ታካሚወች ጋር እኩል ያለአድልኦ የመስተናገድ እድል አግኘተዋል ወይ? **አዎ የለም**
5. በህክምና ምርመራና ማማከር በኩል ምን ያህል ረክተዋል?

በጣም አልረካሁም አልረካሁም ረክቻለሁ በጣም ረክቻለሁ
